# Microglia-Derived Microvesicles Affect Microglia Phenotype in Glioma

**DOI:** 10.3389/fncel.2019.00041

**Published:** 2019-02-22

**Authors:** Alfonso Grimaldi, Carmela Serpe, Giuseppina Chece, Valentina Nigro, Angelo Sarra, Barbara Ruzicka, Michela Relucenti, Giuseppe Familiari, Giancarlo Ruocco, Giuseppe Rubens Pascucci, Francesca Guerrieri, Cristina Limatola, Myriam Catalano

**Affiliations:** ^1^Center for Life Nanoscience, Istituto Italiano di Tecnologia@Sapienza, Rome, Italy; ^2^Department of Physiology and Pharmacology, Sapienza University of Rome, Rome, Italy; ^3^Department of Physics, Istituto dei Sistemi Complessi del Consiglio Nazionale delle Ricerche, Sapienza University of Rome, Rome, Italy; ^4^Department of Science, University of Roma Tre, Rome, Italy; ^5^Department of Anatomical, Histological, Forensic Medicine and Orthopedics Sciences, Sapienza University of Rome, Rome, Italy; ^6^Department of Physiology and Pharmacology, Laboratory Affiliated to Istituto Pasteur Italia – Fondazione Cenci Bolognetti, Sapienza University of Rome, Rome, Italy; ^7^IRCCS Neuromed, Pozzilli, Italy

**Keywords:** microglia, extracellular vesicles, tumor associated myeloid cells, brain tumors, glioma

## Abstract

Extracellular-released vesicles (EVs), such as microvesicles (MV) and exosomes (Exo) provide a new type of inter-cellular communication, directly transferring a ready to use box of information, consisting of proteins, lipids and nucleic acids. In the nervous system, EVs participate to neuron-glial cross-talk, a bidirectional communication important to preserve brain homeostasis and, when dysfunctional, involved in several CNS diseases. We investigated whether microglia-derived EVs could be used to transfer a protective phenotype to dysfunctional microglia in the context of a brain tumor. When MV, isolated from microglia stimulated with LPS/IFNγ were brain injected in glioma-bearing mice, we observed a phenotype switch of tumor associated myeloid cells (TAMs) and a reduction of tumor size. Our findings indicate that the MV cargo, which contains upregulated transcripts for several inflammation-related genes, can transfer information in the brain of glioma bearing mice modifying microglial gene expression, reducing neuronal death and glioma invasion, thus promoting the recovery of brain homeostasis.

## Introduction

Cellular communication has been recently enriched by a new mechanism, that use the cargo transported by extracellular membrane vesicles (EVs). EVs include exosomes (Exo, 10–100 nm diameter), microvesicles (MV, 100–1000 nm) and apoptotic “blebs” (1–2 mm). EVs are produced by all cell types and their production dynamically changes in number and content in response to specific environmental signals. The content of EVs, considered true metabolic units, is released into the cytoplasm of receiving cells, where they mediate several functional effects (Iraci et al., 2017).

In the brain, EVs modulate synaptic activity and neuronal communication (Korkut et al., 2013; Chivet et al., 2014), and also contribute to spreading disease in several CNS pathologies, such as multiple sclerosis (Carandini et al., 2015), Alzheimer’s disease (Aguzzi and Rajendran, 2009; Gouwens et al., 2018), prion disease (Fevrier et al., 2004), and Huntington’s disease (Zhang et al., 2016). Neurocentric vision in acute and chronic diseases of the CNS turned out to be insufficient to explain the mechanisms responsible for several disease onset and progression. The role played by non-neuronal cells, such as astrocytes and microglia, which are in constant communication with neurons to monitor brain parenchyma through their processes, actively contribute to maintain cerebral homeostasis.

EVs released by microglia, similarly to EVs released by macrophages, recapitulate, in their cargo, the inflammatory information of the donor cell (Garzetti et al., 2014; Cunha et al., 2016).

Microglial cells acquire a dysfunctional phenotype in many CNS pathologies, losing their ability to monitor and preserve brain homeostasis. In the context of glioma, tumor associated myeloid cells (TAMs) modify their phenotype and local microenvironment toward a pro-tumor, anti-inflammatory state supporting tumor cell proliferation, survival, and invasion (da Fonseca and Badie, 2013).

In this work, we investigated the role of MV released by polarized microglia on the modulation of microglia state *in vivo*, to verify the hypothesis to use MV to help host microglia to reacquire a homeostatic state in the context of glioma. At this aim EVs, and in particular microvesicles (MV) and exosomes (Exo) released by microglia in inflammatory conditions were isolated and analyzed for their *in vitro* and *in vivo* effects in glioma bearing mice. We demonstrated that *in vitro*, microglia derived LPS/IFNγ-MVs reduced the expression of anti-inflammatory genes in IL4-treated microglia. *In vivo*, LPS/IFNγ-MVs injected in the brain of mice with glioma reduced the anti-inflammatory phenotype of TAMs and significantly reduced tumor size and tumor induced neurotoxicity. We suggest that the cargo of LPS/IFNγ-MV, which contains specific mRNA for inflammatory genes, transfer this information to recipient cells modifying their gene expression profile toward a protective one. Altogether, these findings demonstrate that the administration of exogenous EVs could be a valuable approach to transfer protective signals to TAMs, restoring the homeostatic microglia phenotype.

## Results

### Dimensional and Morphological Analyses of EVs Derived From Microglia

**FIGURE 1:**
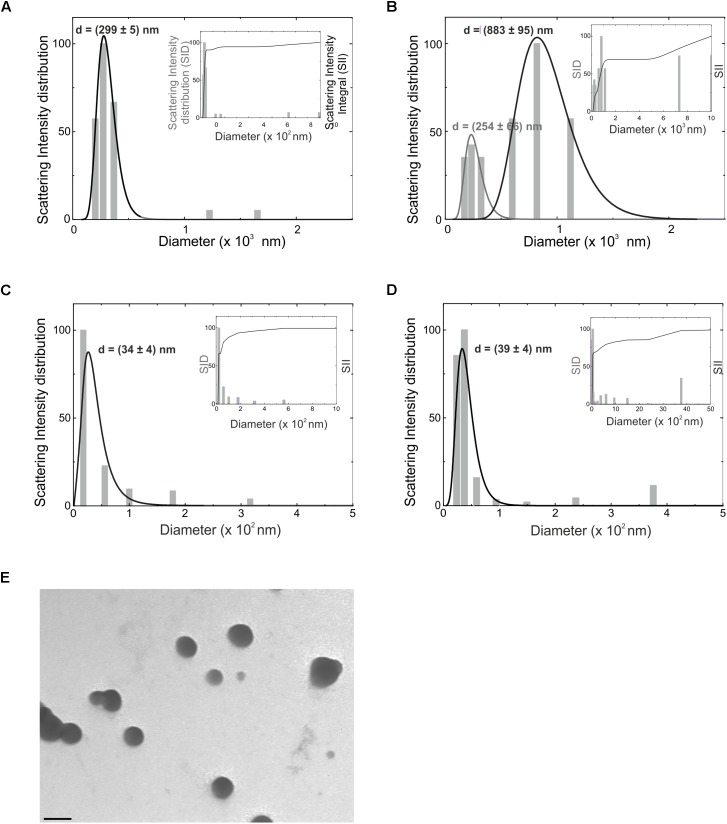
Hydrodynamic diameter distribution (d) as obtained from CONTIN analysis of the intensity autocorrelation curves, for microvesicles obtained from BV2 microglia cell line **(A)** and from primary murine microglia **(B)**, for exosomes from BV2 **(C)** and primary microglia **(D)**, reported as intensity-weighted distribution. The overall normalized integral of the distribution is also reported in the inset of the relative panels (SID, scattering intensity distribution; SII, scattering intensity integral). Gray lines are fits through Log-normal distribution. **(E)** Transmission electron microscopy of EVs derived from BV2 cells. Bar = 100 nm, direct magnification 40000×, print magnification 213000×.

EVs, in particular microvesicles (MV) and exosomes (Exo), were obtained from BV2 cell line and primary mouse microglia. EV sizes were measured by Dynamic Light Scattering (DLS) performed at a constant temperature of 15°C. The CONTIN distribution demonstrated that BV2-derived MV (Figure 1A) had a polydispersity of 20%. The intensity-weighted distribution of hydrodynamic diameter shows that the main population peaked around 300 nm, providing 90% of the total scattered intensity, as evidenced by the integral of the distribution reported in the inset of Figure 1A. In MV released by primary microglia (Figure 1B) a polydispersity of 30–40% was obtained and two main populations were identified: the first peaked at 250 nm and the second at 880 nm. The distribution integral (inset of Figure 1B) highlights that 20% of the total scattered intensity is due to the smallest population, while 50% is due to the largest one. Exo had a polydispersity around 30–40%. Due to their size, the volume-weighted is the most accurate distribution to obtain qualitative information on sample composition. Seventy percent of Exo has a mean size of 34 ± 4 nm (derived from BV2 cells, Figure 1C) and of 39 ± 4 nm (derived from primary microglial cells, Figure 1D), while larger aggregates provide little contribution to the volume-weighted distribution, as evidenced in the insets of Figure 1C,D. For both populations there is a 30% of contribution to the overall scattered intensity due to larger aggregates.

EVs derived from BV2 cells were analyzed at the transmission electron microscopy: data shown in Figure 1E identify the typical round vesicle morphology and underlined a scattered composition in size, with the presence of aggregates (Figure 1E), that confirmed DLS data above reported.

### MV Derived From Microglia Treated With LPS/IFNγ Reduce Migration and Invasion of GL261 Glioma Cells and Are Neuroprotective Against Glioma Excitotoxicity *in vitro*

**FIGURE 2:**
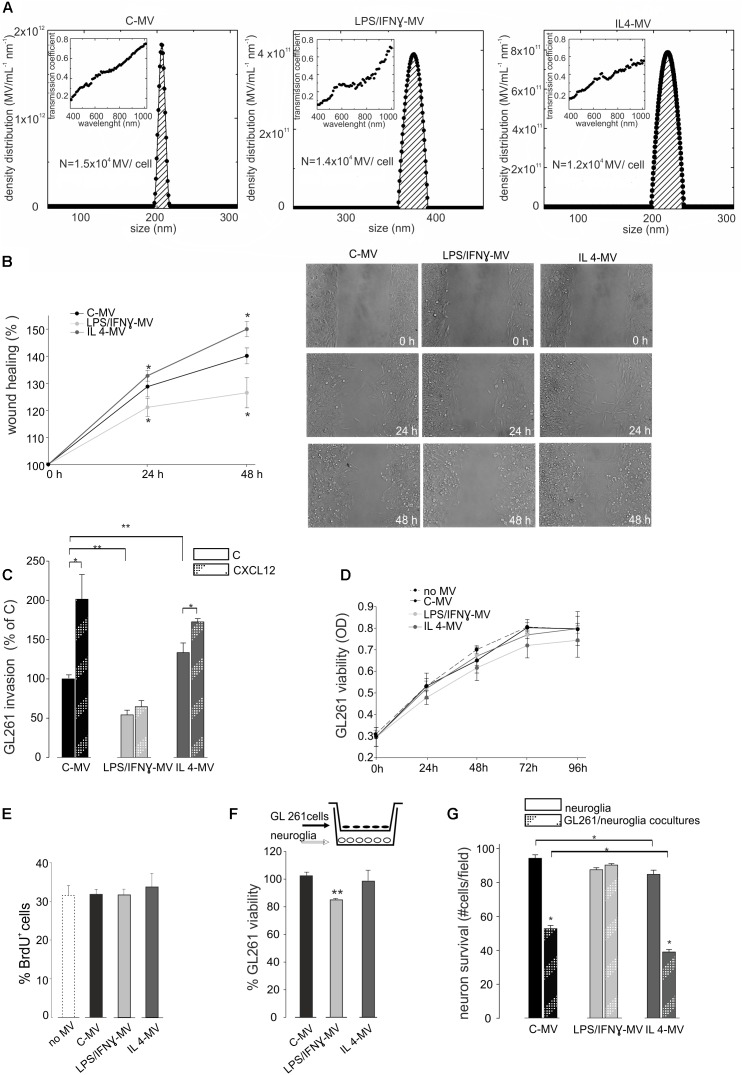
**(A)** Density distribution of BV2-derived MVs, together with the transmission coefficient as a function of the wavelength (inset). The integral of the density distribution showed the number of MV per ml. Normalizing, the number of vesicles produced by each cell in control condition is *N* = 1.5 × 10^4^ (C-MV), with LPS/IFNγ is *N* = 1.4 × 10^4^ (LPS/IFNγ-MV), with IL4 is *N* = 1.2 × 10^4^ (IL4-MV). **(B)** GL261 glioma cells were treated with MV obtained from untreated microglia (C-MV), microglia treated with LPS/IFNγ (LPS/IFNγ-MV) and IL 4 (IL 4-MV) and a wound healing assay was performed. GL261 migration was measured 24 and 48 h after treatment, data are expressed as mean percentage of wound healing area ± SE, *N* = 4, ^∗^*P* < 0.001 vs. C-MV (one way analysis of variance, Holm-Sidak method). On the right, representative wound healing assay on GL261 cells at 0 h and after 24 and 48 h of C-MV, LPS/IFNγ-MV and IL 4-MV treatment. **(C)** GL261 cells were assayed for basal invasion **(C)** and CXCL12-induced invasion in presence of MV obtained from untreated microglia (C-MV), microglia treated with LPS/IFNγ (LPS/IFNγ-MV) and IL 4 (IL 4-MV) in a Boyden chamber system. GL261 invasion was measured as mean percentage ± SE, *N* = 4; ^∗^*P* < 0.05 and ^∗∗^*P* < 0.001 (one way analysis of variance, Holm-Sidak method). **(D)** GL261 cells were assayed for cell viability without MV (no-MV), in presence of MV obtained from untreated microglia (C-MV), microglia treated with LPS/IFNγ (LPS/IFNγ-MV) and IL 4 (IL 4-MV) by MTT analysis. Cells were analyzed 0, 24, 48, 72, and 96 h after plating. Viability was reported in optical density (OD) mean ± SE, *N* = 4; no statistical significance vs. C-MV (one way analysis of variance, Holm-Sidak method). **(E)** GL261 cell proliferation was measured as % mean ± SE of BrdU^+^ GL261 cells untreated (no-MV), treated with MV obtained from untreated microglia (C-MV), microglia treated with LPS/IFNγ (LPS/IFNγ-MV) and IL 4 (IL 4-MV) for 24 h, *N* = 4, no statistical significance vs. C-MV (one way analysis of variance, Holm-Sidak method). **(F)** GL261 cells, co-cultured with hippocampal neuroglial cultures (as depicted in the inset) were treated for 18 h with MV obtained from untreated microglia (C-MV), microglia treated with LPS/IFNγ (LPS/IFNγ-MV) and IL 4 (IL 4-MV), were analyzed for viability by trypan Blue staining. Data are expressed as number of viable cells/field ± SE, *N* = 3; ^∗∗^ *P* < 0.05, Student’s *t*-test. **(G)** Hippocampal neuroglial cultures alone or co-cultured with GL261 cells (as depicted in the inset of panel **F**) were treated with MV obtained from untreated microglia (C-MV), microglia treated with LPS/IFNγ (LPS/IFNγ-MV) and IL 4 (IL 4-MV) for 18 h and were analyzed for neuronal viability. Data are expressed as number of viable cells/field ± SE, *N* = 3, ^∗^*P* < 0.001, Student’s *t*-test.

Cultured primary microglia and BV2 cells were treated with LPS/IFNγ or IL 4. We previously verified microglia polarization by mRNA analysis (Grimaldi et al., 2016); we confirmed these data showing that LPS/IFNγ-treated cells increased NO release and that IL-4-treated cells increased Arg1 expression and activity (Supplementary Figure S1 and related Supplementary Methods). Quantification of MV released by control (C-MV), LPS/IFNγ-treated (LPS/IFNγ-MV), and IL4-treated BV2 cells (IL4-MV) was measured by Laser Transmission Spectroscopy (LTS) (Li et al., 2010). LTS measurements showed that the number (N) of MV/cell in these three conditions was not significantly different (Figure 2A). MV were then tested to verify their ability to interfere with glioma cell migration and proliferation. Data shown in Figure 2B illustrate that GL261 migration was impaired by LPS/IFNγ-MV, while GL261 migration increased upon IL 4-MV treatment, at 24 and 48 h. In contrast, neither LPS/IFNγ-Exo nor IL 4-Exo (released by primary microglia) affected GL261 migration (Supplementary Figure S2). Based on these results, for successive experiments we decided to focus our interest only on the effect of MV. Similarly to migration, results obtained on CXCL12-induced GL261 invasion, demonstrated inhibitory effects of LPS/IFNγ-MV and no effects of IL 4-MV (Figure 2C). Note that basal GL261 invasion was significant reduced in the presence of LPS/IFNγ-MV and was significant enhanced in the presence of IL 4-MV. These data indicate that microglia-derived MVs modulate glioma cell movement and invasion.

To investigate the effect of MV on glioma cell viability and proliferation, MTT assay and BrdU staining were performed. Results reported in Figure 2D show that neither LPS/IFNγ-MV nor IL 4-MV directly modulate GL261 viability, at 24, 48, 72, or 96 h. Similarly, no changes in proliferation rate were induced by LPS/IFNγ-MV or by IL 4-MV, measured by bromodeoxyuridine (BrdU) staining after 24 h (Figure 2E) and 48 h (data not shown). To investigate whether microglia-derived MV could exert indirect effects on glioma, cells were co-cultured with a mixed neuroglia culture in the presence of LPS/IFNγ-MV, for 18 h: in these conditions a reduction of GL261 viability was observed (Figure 2F), indicating an indirect effect of MV on glioma cell viability. In these same co-culture experiments, we also analyzed the effect of MV on neuron survival and observed (Figure 2G) that LPS/IFNγ-MV counteracted GL261-induced neurotoxicity, while IL 4-MV were ineffective.

### Microglia-Derived LPS/IFNγ-MV Reduce Tumor Size in Mice

**FIGURE 3:**
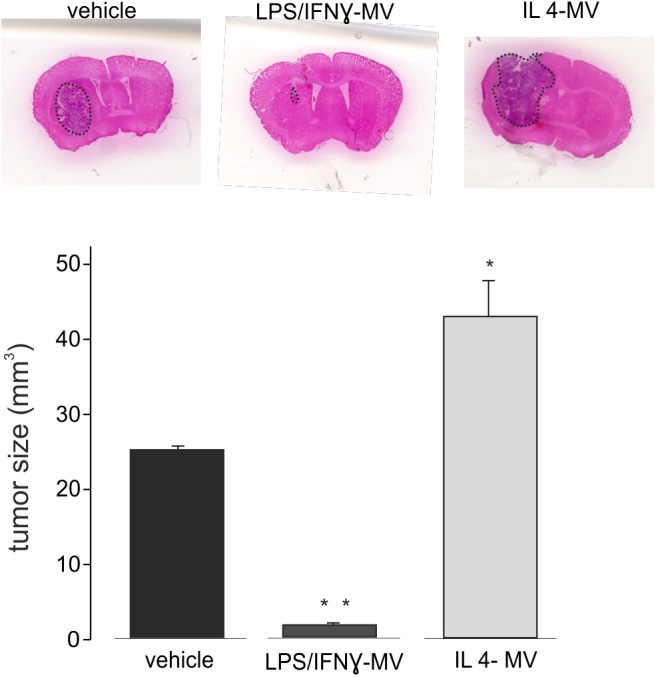
Tumor size in the brain of GL261-bearing mice treated with vehicle or MV obtained from microglia treated with LPS/IFNγ (LPS/IFNγ-MV) and IL 4 (IL 4-MV). Tumor size (in mm^3^) was reported as mean ± SE, *N* = 7/experimental group, ^∗^*P* < 0.05, ^∗∗^*p* ≤ 0.001 vs. vehicle, Student’s *t*-test. On the top, representative coronal brain sections of GL261-bearing mice treated as above, stained with hematoxylin-eosin; tumor area in the dashed line.

**FIGURE 4:**
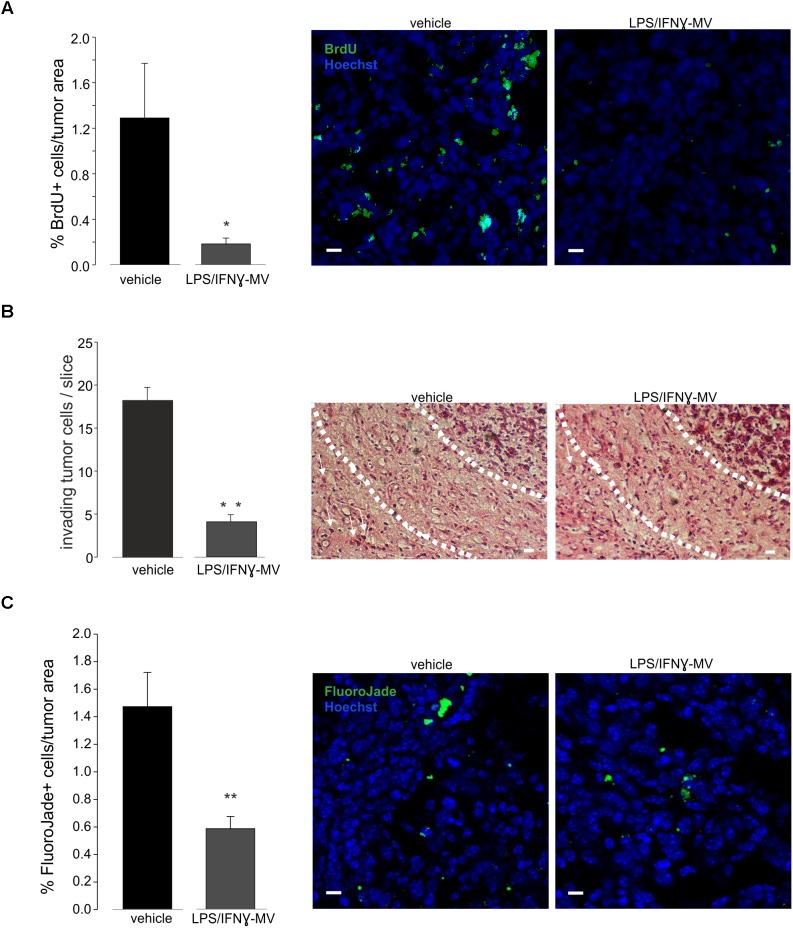
**(A)** Cell proliferation, measured as BrdU^+^ cell area/tumor area in GL261-bearing mice treated with vehicle or MV obtained from microglia treated with LPS/IFNγ (LPS/IFNγ-MV), data are expressed as mean percentage ± SE, *N* = 3, ^∗∗^*P* ≤ 0.001 vs. vehicle, Student’s *t*-test. On the right: representative immunofluorescence on coronal brain slices of GL261-bearing mice treated as above (BrdU in green; Hoechst in blue), scale bars, 20 μm. **(B)** Glioma cells invading the brain parenchyma for more than 150 μm beyond tumor border 17 days after glioma cell transplantation in GL261-bearing mice treated with vehicle or MV obtained from microglia treated with LPS/IFNγ (LPS/IFNγ-MV), data are expressed as mean cell number ± SE, *N* = 3/experimental group, ^∗∗^*P* ≤ 0.001, Student’s *t*-test vs. vehicle. On the right, representative brain peritumoral sections stained with haematoxylin/eosin; white arrows indicate glioma cells invading the brain parenchyma beyond the main tumor border for more than 150 μm (dashed line); scale bars, 20 μm. **(C)** Neuronal death, measured as Fluoro Jade^+^ cells/tumor area in GL261-bearing mice treated with vehicle or MV obtained from microglia treated with LPS/IFNγ (LPS/IFNγ-MV), data are expressed as mean percentage ± SE, *N* = 3, ^∗∗^*P* ≤ 0.001 vs. vehicle, Student’s *t*-test. On the right: representative immunofluorescence on coronal brain slices of GL261-bearing mice treated as above (Fluoro Jade in green; Hoechst in blue), scale bars, 20 μm.

The above reported data prompted us to investigate the effect of LPS/IFNγ-MV in a mouse model of glioma. At this aim, GL261 cells were brain injected in the striatal region of the right hemisphere and, after 7 and 14 days, primary microglia-derived MV were infused in the tumor region via an implanted cannula. Fifteen days after tumor injection (Figure 3) LPS/IFNγ-MV treated mice had a significant reduction of tumor size while mice treated with IL 4-MV significantly increased the size of their brain tumors. Similar results were obtained with BV2-derived MV (Supplementary Figure S3). To investigate whether these effects on tumor size were mediated by alterations of GL261 cell proliferation, mice treated as in Figure 3 were given BrdU 2 h before euthanasia. Quantification of BrdU-labeled cells, normalized for tumor area, revealed a significant reduction of cell proliferation in the tumoral region (Figure 4A) of LPS/IFNγ-MV treated mice. Treatment with LPS/IFNγ-MV also reduced glioma cell migration *in vivo*, as reported in Figure 4B, where tumor cell invasion was calculated as the number of cells protruding more than 150 μm from the main tumor mass. In the peritumor region, we evaluated neuronal death by Fluoro Jade staining, comparing mice treated with LPS/IFNγ-MV with control mice. Data shown in Figure 4C demonstrate that neuronal death was significant reduced in LPS/IFNγ-MV treated mice, demonstrating *in vivo* a neuroprotective effect of LPS/IFNγ-MV against tumor-induced excitotoxicity.

All these data demonstrated that LPS/IFNγ-MV carry anti-tumor information that are effective in reducing tumor size *in vivo*.

### Microglia-Derived LPS/IFNγ-MVs Modify the Phenotype of Tumor-Associated Microglia

**FIGURE 5:**
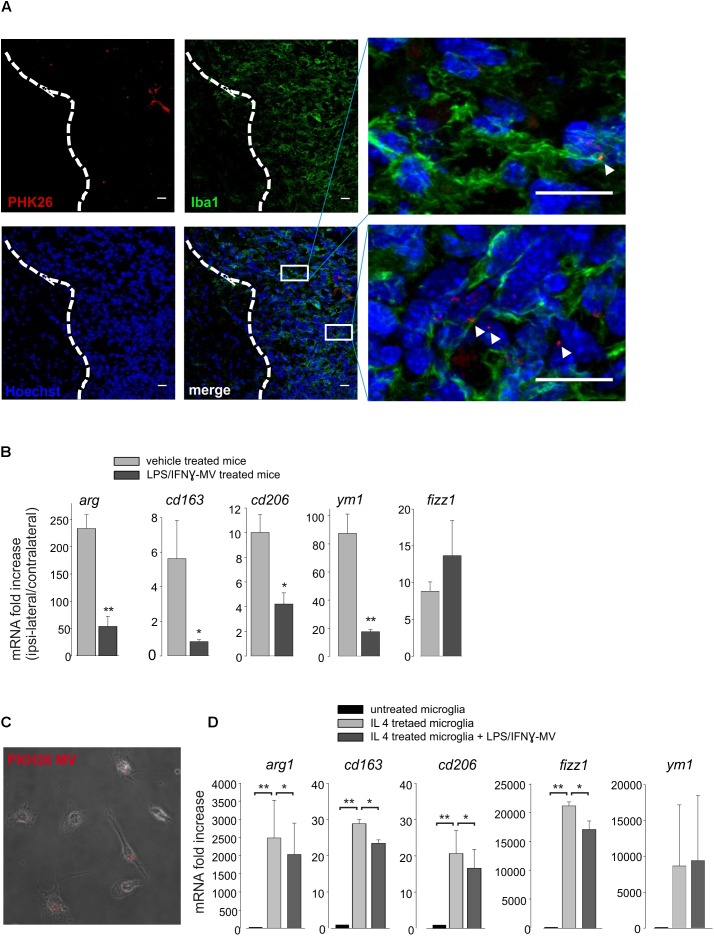
**(A)** Immunofluorescence analysis for Iba1 (in green) of coronal brain sections from GL261-bearing mice treated with PKH26-stained MV (in red; Hoechst in blue) obtained from microglia. Tumor region at the right of the dashed line. In the merge, identification of two tumor regions shown at higher magnification. Scale bar: 20 μm **(B)** RT-PCR of anti-inflammatory genes (*arg1*, *cd163*, *cd206*, *ym1*, and *fizz1*) in CD11b^+^ cells extracted from brains of GL261-bearing mice treated with vehicle or MV obtained from microglia treated with LPS/IFNγ (LPS/IFNγ-MV). Data are the mean ± SE of fold increase (normalized vs. contralateral cerebral hemisphere of each animal; gene expression was normalized vs. *gapdh*), *N* = 5, ^∗∗^*P* < 0.001, ^∗^*P* < 0.05 vs. vehicle treated mice, Student’s *t*-test. **(C)** Merged of bright and fluorescence fields of microglia treated with IL 4 and incubated with PKH26-stained MV (in red) obtained from LPS/IFNγ-treated microglia. **(D)** RT-PCR on mRNAs of untreated or IL 4-treated microglia incubated with or without MV obtained from LPS/IFNγ-treated microglia, and analyzed for the expression of anti-inflammatory genes (*arg1*, *cd163*, *cd206*, *fizz1*, and *ym1*). Data are the mean ± SE of fold increase (normalized vs. *gapdh*), *N* = 3, ^∗∗^*P* < 0.001, ^∗^*P* < 0.05, Student’s *t*-test.

To understand the mechanisms of action of the injected MVs, we first decided to investigate their cellular localization upon brain injection. At this aim, MVs derived from primary microglia, were stained with the membrane-selective dye PKH26 and brain injected in glioma bearing mice. Cerebral slices obtained from the tumoral region show the presence of PKH26-MV on both Iba1^-^ and Iba1^+^ cells (Figure 5A). We never observed PKH26 staining outside the tumor area. It is known that a high amount of tumor associated microglia/macrophages (TAMs) are present in glioma, and may represent up to 50% of tumor mass (Hambardzumyan et al., 2016). TAMs exert tumor supporting function releasing factors that facilitate tumor proliferation and migration (Fonseca et al., 2012). We wonder whether the interaction of MVs with TAMs could modulate their pro-tumor activity, and investigated the effect of LPS/IFNγ-MV on gene expression of CD11^+^ cells isolated from the brains of glioma-bearing mice. As shown in Figure 5B, the expression of *arg1*, *cd163*, *cd206*, *fizz1*, and *ym1* genes all correlated with an anti-inflammatory, pro-tumor phenotype, were up-regulated in CD11b^+^ cells in the ipsilateral hemisphere of glioma bearing mice and were significant reduced upon LPS/IFNγ-MV treatment (with the exception of *fizz1*). Similar modulatory effects were observed *in vitro*: as shown in Figure 5D, LPS/IFNγ-MV (derived from primary microglia) significant reduced the microglial expression of all analyzed genes (*arg1*, *cd163*, *cd206*, *ym1*, and *fizz1*) except *ym1*, indicating a direct modulation likely induced by MV cargo that enter microglia also *in vitro* (Figure 5C).

All these data suggest that LPS/IFNγ-MVs might directly signal to microglia and instruct these cells toward an antitumor phenotype.

### mRNA Analyses of Microglia-Derived LPS/IFNγ-MV

**FIGURE 6:**
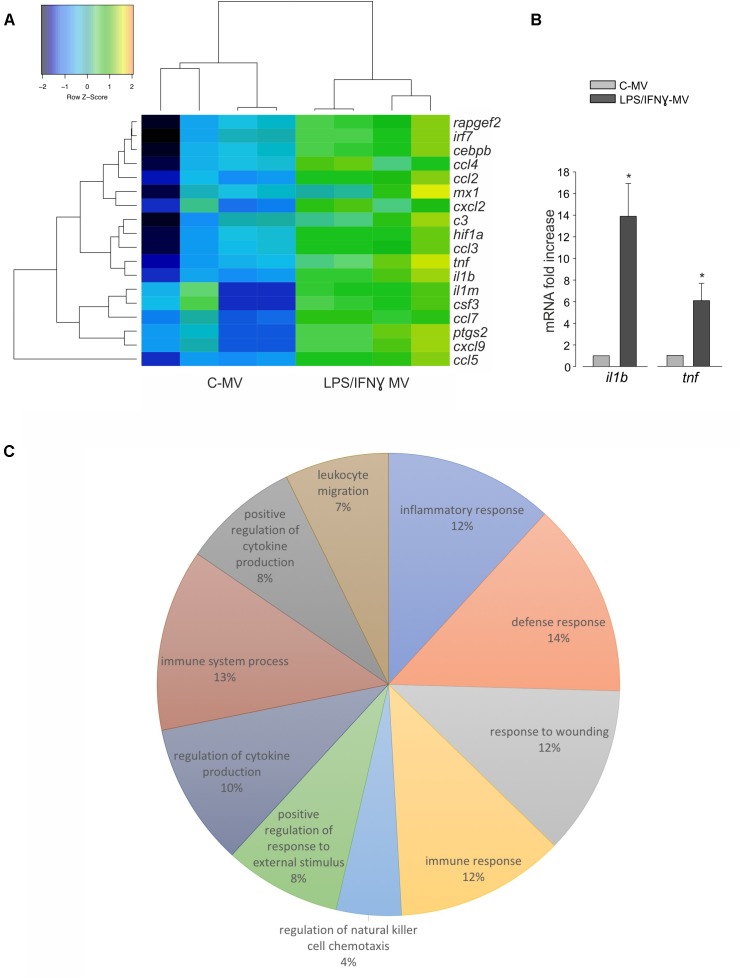
**(A)** mRNAs isolated from MV released by untreated microglia (C-MV) or microglia treated with LPS/IFNγ (LPS/IFNγ-MV) were analyzed by NanoString. Heat map analysis of NanoString data show hierarchical clustering of 18 differentially expressed genes between C-MV and LPS/IFNγ-MV, *N* = 4. The signals were normalized and transformed to the log2 scale. The color scale indicates the gene expression standard deviations from the mean, with black/blue for low expression and green/orange for the high expression levels. Eighteen genes (listed on the right) were considered significant because with at least 2.8-fold changes with *P* < 0.05 (Wilcoxon test) at the 95% confidence level. Dendrograms illustrate the relationship-distance between samples and genes. **(B)** RT-PCR on mRNAs isolated from MV derived from untreated microglia (C-MV) or microglia treated with LPS/IFNγ (LPS/IFNγ-MV) analyzed for the expression of *il1b* and *tnf* genes. Data are the mean ± SE of fold increase (normalized vs. *gapdh*), *N* = 3, ^∗^*P* < 0.05, Student’s *t*-test vs. C-MV. **(C)** The pie chart illustrates the distribution of the 18 differentially expressed genes across the 10 most significant functional categories defined by the Gene Ontology Biological process. The percentage numbers represent the frequency of genes in each category. Data were analyzed using FIDEA tool.

To investigate the nature of the cargo transported by LPS/IFNγ-MV that could help the understanding of the above described mechanisms, the mRNA content of MVs released by primary microglia was analyzed by NanoString chip for the expression of 243 key inflammation-related genes and compared with MVs released by control, unstimulated microglia. As shown on Figure 6A, 18 genes were significantly up-regulated in LPS/IFNγ-MV. Data were confirmed by RT-PCR for *tnf*-α and *il1b* genes (Figure 6B), validating the immune panel system.

Pathway analysis, performed on the 10 most significant functional categories defined by the Gene Ontology Biological process (Figure 6C) indicated that LPS/IFNγ-MV contain 12% of genes involved in inflammatory responses (*c3*, *ccl4*, *ccl5*, *cxcl2*, *cxcl9*, *tnf*, *ptgs2*, *ccl7*, *il1b*, *ccl2*, *ccl3*, *il1rn*, *hif1a*), 14% of genes involved in defense responses (*irf7*, *c3*, *ccl4*, *ccl5*, *cxcl2*, *cxcl9*, *tnf*, *ptgs2*, *mx1*, *ccl7*, *il1b*, *ccl2*, *ccl3*, *il1rn*, *hif1a*), 12% of genes involved in responses to wounding (*c3*, *ccl4*, *ccl5*, *cxcl2*, *cxcl9*, *tnf*, *ptgs2*, *ccl7*, *il1b*, *ccl2*, *ccl3*, *il1rn*, *hif1a*), 12% of genes involved in immune responses (*irf7*, *c3*, *ccl4*, *ccl5*, *cxcl2*, *cxcl9*, *tnf*, *mx1*, *ccl7*, *il1b*, *ccl2*, *ccl3*, *csf3*), 4% of genes involved in regulation of natural killer cell chemotaxis (*ccl7*, *ccl4*, *ccl3*, *ccl2*, *ccl5*), 8% of genes involved in positive regulation of response to external stimuli (*c3*, *ccl4*, *ccl5*, *tnf*, *ccl7*, *ptgs2*, *il1b*, *ccl2*, *ccl3*), 10% of genes involved in regulation of cytokine production (*irf7*, *c3*, *ccl4*, *ccl5*, *tnf*, *ptgs2*, *il1b*, *ccl2*, *ccl3*, *hif1a*, *cebpb*), 13% of genes involved in immune system process (*irf7*, *c3*, *ccl4*, *ccl5*, *cxcl2*, *cxcl9*, *tnf*, *mx1*, *ccl7*, *il1b*, *ccl2*, *ccl3*, *hif1a*, *csf3*), 8% of genes involved in positive regulation of cytokine production (*irf7*, *c3*, *ccl4*, *ccl5*, *tnf*, *ptgs2*, *il1b*, *ccl3*, *hif1a*), and 7% of genes involved in leukocyte migration (*ccl4*, *ccl5*, *cxcl2*, *tnf*, *ccl7*, *il1b*, *ccl2*, *ccl3*). These data demonstrated that the MV mRNA content is highly complex, covering genes coding for proteins related to different pathways of the inflammatory process, and that their delivery to TAM might contribute to re-activate the immune response, which oppose glioma growth.

## Materials and Methods

### Primary Brain Cells Cultures

Neuroglial cultures were prepared from 0- to 2-day-old (p0–p2) C57BL/6N mice as already described (Lauro et al., 2010); neurons-astrocytes-microglia ratio is 60-35-5%. Microglia cultures were obtained as already reported (Grimaldi et al., 2016) and were 98% Iba1^+^ positive (Lauro et al., 2010).

### Cell Lines

BV2 murine microglial cells and GL261 murine glioma cells were cultured in DMEM supplemented with 10–20% heat-inactivated FBS, 100 IU/ml penicillin G, 100 μg/ml streptomycin, 2.5 μg/ml amphotericin B and grown at 37°C in a 5% CO_2_ and humidified atmosphere.

### Microglia Stimulation (Polarization)

Primary microglia and BV2 cells were treated for 24 hours with IFN-γ (20 ng/ml) and LPS (100 ng/ml), or with IL-4 (20 ng/ml) or with EVs.

### Extraction of EVs

Cytokine-treated microglia were stimulated for 30 min with ATP (1 mM) in KRH (125 mM NaCl; 5 mM KCl; 1.2 mM MgSO_4_; 1.2 mM KH_2_PO_4_; 2 mM CaCl_2_; 6 mM D-glucose; 25 mM HEPES/NaOH, pH = 7.4). Cell supernatant was collected and centrifuged at 800 *g* for 5 min to remove cell debris. The obtained supernatant was centrifuged at 10000 *g* for 30 min at 4°C and the resulting pellet, containing MVs, was re-suspended in KRH buffer for DLS, LTS and transmission electron microscopy, in PBS for *in vivo* experiments or in serum-free DMEM for microglia stimulation, MTT, wound healing and BrDU proliferation assays. Remaining supernatant underwent ultracentrifugation at 100,000 *g* for 1-h at 4°C. The Exo, present in this second pellet, were re-suspended in KRH buffer for DLS measures and in serum free DMEM for wound healing assay. The same protocol permits to eliminate EVs from the medium used to re-suspend MVs and Exo and guarantees that the EVs obtained only derived from polarized microglial cells. In some experiments, EVs were labeled with PKH26, a lipophilic membrane red fluorescent dye (PKH26 GL-1KT, Sigma-Aldrich) according to manufacturer protocol. Briefly, PKH26 dye was re-suspended in diluent C to a final concentration of 0.6 μM (dye solution). MV were re-suspended in 500 μL of dye solution and incubated for 5 min, while mixed with gentle pipetting. Excess dye was bound with 500 μL EVs-depleted bovine serum albumin (1%, Sigma-Aldrich). MV pellet, obtained by centrifugation (10000 × *g*, 30 min, 4°C) was washed twice in PBS. PKH26-stained MV were re-suspended in PBS for *in vivo* administration and in serum-free DMEM for *in vitro* treatment. In all the experiments, the same procedure of staining minus MV was performed as control condition.

### Dynamic Light Scattering and Data Analysis

Dynamic light scattering (DLS) measurements were performed using a standard optical setup. The monochromatic and polarized beam emitted from a He-Ne laser (10 mW at λ = 632.8 nm) was focused on the sample placed in a capillary of 2 mm of diameter positioned in a cylindrical VAT for index matching and temperature control. The scattered intensity was collected at a scattering angle θ = 90°that, according to the relation Q = (4πn/λ) sin(θ/2), corresponds to a scattering vector Q = 0.0187 nm^-1^. A single mode optical fiber collected the scattered light as a function of time and the signal was detected by a photomultiplier. In this way the normalized intensity autocorrelation function g_2_(Q,t) = < I(Q,t)I(Q,0)>/<I(Q,0) >^2^ with a high coherence factor close to the ideal unit value was measured. Measurements were performed at fixed temperature around 15°C. Reproducibility has been tested by repeating measurements several times on different samples. The intensity correlation curves obtained from DLS experiments have been analyzed with the Laplace inversion through the CONTIN algorithm weighted as overall contribution to the scattered intensity. However, the scattered intensity depends on the squared volume of the scattering particle, thus leading to an overestimation of the large particles. Therefore, this approach has good reliability for samples of EVs, whose hydrodynamic diameter is expected in the range D_h_ = 100÷1000 nm. On the other hand, small particles are under-represented in the intensity-weighted distribution and Laplace transformation can be very inaccurate. To represent the hydrodynamic diameter distribution of Exo with size expected in the range D_h_ = 10÷100 nm the volume-weighted distribution has been chosen. The mean size of each population has been calculated by fitting the scattering intensity distribution with a Log-normal function.

### Laser Transmission Spectroscopy

Laser Transmission Spectroscopy (LTS) allows to obtain the density of MV in suspension by measuring the transmission of laser light (at zero angle with respect to the incoming beam) through the suspension as a function of wavelength (Li et al., 2010). The transmission of light through the MV sample (re-suspended in KRH) is recorded along with that of KRH. The fundamental data-acquisition process involves measuring the wavelength-dependent transmission of light through an aqueous suspension of vesicles. Given the extinction information, and the known wavelength-dependent properties of the vesicles, Mie theory can be used to accurately determine their density distribution as a function of diameter. The extinction data are analyzed and inverted by a mean square root-based algorithm that outputs the particle size distribution. The integral of the density distribution provides the number of MV per ml of solution and the absolute number (N) of MV per cell is calculated as the ratio between the number of MV in a given volume and the total number of MV donor cells.

### Transmission Electron Microscopy

Microvesicles derived from BV2 cells (extracted as above) were fixed by glutaraldehyde 2.5% in PBS buffer pH 7.4 at least 2 h and gently resuspended in PBS buffer. A drop of the solution was put on a Formvar copper grid 200 mesh for 10 min. After drying with filter paper, uranyl acetate aqueous solution (1%, 1 min) was used for negative staining. Samples were observed with a ZEISS EM 10 transmission electron microscope equipped with a Gatan CCD camera at 60 kw.

### Neuronal Viability Assay

Neuroglial cultures at 9–11 days *in vitro* were co-cultured with GL261 cells (5 × 10^4^/well) in the presence or absence of EVs with a ratio of donor:target cells 1:1. After 18 h, cells were treated with detergent-containing buffer and counted in a haemocytometer as already described (Lauro et al., 2010).

### MTT Cell Viability Assay

GL261 cells were seeded into 24-well plates and treated with vehicle (untreated), IFN-γ /LPS- or IL 4-MV for 3 days. MTT (500 μg/ml) was added into each well for 1.5 h. DMSO was then added to stop the reaction and the formazan produced was measured at 570 nm. Viability of cells was expressed relative to absorbance.

### Wound-Healing Assay

GL261 cells (5 × 10^5^/ml) were seeded into the inner wells of cell culture inserts (ibidi, Germany) placed in a Petri dish. Once attached to the substratum, the inserts were removed, leaving a central 500 μm cell-free septum in which cells could migrate. Cell medium with MV released by IFN-γ /LPS- or IL 4-treated microglia (ratio 1:1 = donor: target cells) was added. Cells were incubated with a cell cycle blocker (cytarabine, 10 μM) to prevent GL261 proliferation for all the time of the experiment. Dishes were maintained at 37°C, 5% CO_2_. Pictures of the starting point (0 h) and 24 and 48 h after treatment were taken at a phase contrast microscope (Nikon) and processed through MetaMorph 7.6.5.0 software (Molecular Device). GL261 migration was evaluated by the area between the two cell fronts (by ImageJ software) and data are expressed as % of area occupied by cells.

### Invasion Assay

GL261 cells (7 × 10^3^ cells/cm^2^) were plated on matrigel-coated polycarbonate membranes (8 μm diameter pores, Corning) of a Boyden Chamber system in presence of IFN-γ /LPS-MV or IL 4-MV (ratio 1:1 donor:target cells) and incubated for 48 h at 37°C with CXCL12 (100 nM, Peprotech) in the lower chamber as chemoattractant. The experiments were performed in presence of cytarabine (10 μM). Cells adhering to the upper side of the membranes were scraped off, whereas cells that have invaded through the pores were stained with a solution containing 50% isopropanol, 1% formic acid, and 0.5% (wt/vol) brilliant blue R 250 and counted in more than 20 fields with a 20× objective.

### *In vivo* Experiments

Experiments were approved by the Italian Ministry of Health, in accordance with the ethical guidelines on use of animals from the EC Council Directive 2010/63/EU. Eight-week-old male C57BL/6N mice were injected with GL261, as previously described (Grimaldi et al., 2016), in the right striatal brain region. During surgery, a guide cannula was placed 2 mm deep in the striatum and it was fixed with quick-setting cement. After 7 and 14 days, mice were infused via cannula with MVs obtained from 1 × 10^6^ microglia cells re-suspended in 4 μl PBS). The day after the second infusion, animals were sacrificed and analyzed for tumor size (Grimaldi et al., 2016). Alternatively, mice were deeply anesthetized and CD11b^+^ cells were isolated as already described (Grimaldi et al., 2016). Obtained cells were lysed in Trizol reagent (Invitrogen, Milan, Italy) for RNA extraction and Real Time PCR analysis.

### BrdU Proliferation Assay

GL261 cells were grown on glass coverslips (1.5 × 10^4^ cells/cm^2^) in 24-well plates for 18 h. Cells were exposed to vehicle or EV for 24 h, cellular proliferation was analyzed adding BrdU (10 μM, Sigma-Aldrich, B5002) for 30 min. Cells were washed in PBS, fixed (4% PFA, 30 min), permeabilized (1% Triton X-100, 15 min), blocked (1% BSA, 1 h) at RT and incubated overnight with anti-BrdU (1:200, Novusbio, NB500-169). Hoechst was used to stain all nuclei. BrdU positive cells were counted out of 800 cells for condition. Proliferation rate is calculated as BrdU^+^ cells respect to Hoechst stained cells. *In vivo*, 15 days after glioma cell injection, BrdU was i.p. injected (50 mg/kg). Two hours later, mice were killed and their brains processed for immunofluorescence.

### Immunofluorescence

Coronal brain sections (20 μm) were washed in PBS, blocked (3% goat serum in 0.3% Triton X-100) for 1h at RT and incubated with anti-Iba1 (1:500, Wako, 019-19741, 4°C) or anti-BrdU (1:200, Novusbio, NB500-169, RT). Brain slices were stained with the fluorophore-conjugated secondary antibodies (1 h, RT) and Hoechst for nuclei visualization and analyzed using a fluorescence microscope. For Fluoro Jade-C staining, we followed the manufacturer instructions (Millipore, AG325).

### Real Time PCR

RNAs extracted from all samples were quantified and retro-transcribed using IScriptTM Reverse Transcription Supermix (Bio-Rad). Real time PCR (RT-PCR) was carried out in an I-Cycler IQ Multicolor RT-PCR Detection System (Bio-Rad) using SsoFast Eva Green Supermix (Bio-Rad). The PCR protocol consisted of 40 cycles of denaturation at 95°C for 30 s and annealing/extension at 58°C for 30 s. The Ct values from each gene were normalized to the Ct value of GAPDH. Relative quantification was performed using the 2^-ΔΔCt^ method and expressed as fold increase. Primer sequences: arg1, forward: CTCCAAGCCAAAGTCCTTAGAG, reverse: AGGAGCTGTCATTAGGGACATC; cd163 forward: GCTAGACGAAGTCATCTGCACTGGG, reverse: TCAGCCTCAGAGACATGAACTCGG; cd206 fw: CAAGGAAGGTTGGCATTTGT, reverse: CCTTTCAGTCCTTTGCAAGT; ym1 forward: CAGGTCTGGCAATTCTTCTGAA, reverse: GTCTTGCTCATGTGTGTAAGTGA; fizz1 forward: CCAATCCAGCTAACTATCCCTCC, reverse: ACCCAGTAGCAGTCATCCCA; gapdh forward: TCGTCCCGTAGACAAAATGG, reverse: TTGAGGTCAATGAAGGGGTC.

### RNA Isolation and NanoString nCounter Analysis

Total RNA was isolated from microglia-derived MV by Total Exosome RNA Protein Isolation Kit (# 4478545, Invitrogen) and concentrated using the Microcon10 centrifugal filters (#MRCPRT010, Merck Millipore). Gene expression raw data were normalized considering housekeeping genes via nSolver Software (NanoString). Statistical analysis was conducted in R (version 3.5.0) with RStudio (version 1.1.383^1^). On normalized and log2-transformed data, differential expression was tested applying the Wilcoxon test (*P* < 0.05) and filtering by log2FC > 1.5. All differentially expressed genes were classified into several catalogs according to the Gene Ontology (GO) annotation. The over-representation analyses of GO terms, including biological process and molecular function, were performed using the FIDEA tool^2^.

### Statistical Analysis

Statistical analyses were performed using SigmaPlot 11.0 Software unless otherwise stated.

## Discussion

The present study demonstrated that microglia-derived LPS/IFNγ-MV transfer a protective-antitumor phenotype to the brain of glioma bearing mice. We demonstrated that LPS/IFNγ-MV, which contain the transcripts for a number of inflammation-related genes, can modify TAMs phenotype reducing the expression of anti-inflammatory genes, exert protective effects on neurons and reduce glioma cell proliferation and invasion in surrounding parenchyma.

We have shown that LPS/IFNγ-MV contain 18 genes upregulated in comparison with MV isolated by unstimulated microglia. The majority of these genes referred to the immune response that could underlie the robust effect of MV on the modification of microglia phenotype. It is known that many solid tumors, such as colon-rectal, epithelial ovarian, brain and lung cancers, release EVs which are involved in supporting TAM reprogramming toward an anti-inflammatory, tumor-supporting phenotype (Fabbri et al., 2012; Challagundla et al., 2015; Neviani and Fabbri, 2015; Chen et al., 2017; Shinohara et al., 2017; Cooks et al., 2018). Our findings demonstrated that in the context of a brain tumor, microglia-derived MV can be used in reverse, as a tool to contrast glioma progression, modulating local microenvironment.

Previous reports described that EVs released from LPS stimulated BV2 cell line contain cytokines such as TNF-α and IL-6, as well as ribosome, focal adhesion, extracellular matrix, and membrane proteins (Yang et al., 2018). BV2-derived EVs expressing the cytokine IL-4, delivered in a mouse model of multiple sclerosis, propagated an anti-inflammatory response, upregulating microglia and macrophage expression of chitinase 3-like 3 (ym1) and arginase (arg-1), which reduced neuroinflammation with protective effects on tissues (Casella et al., 2018). Consistently, we observed that the EVs released by cytokine-stimulated microglia maintain the phenotype of the donor cells. This result is in line with the evidence that vesicles released by macrophages and dendritic cells reflect the inflammatory state of original cells (Kim et al., 2005; Viaud et al., 2009; Garzetti et al., 2014).

Microvesicle are supposed to transfer their cargo by docking at the plasma membrane of target cells, nevertheless the exact mechanism is not fully revealed. This interaction is neither stochastic nor unspecific because MV do recognize the target cells (Losche et al., 2004). The recognition takes place by activation of specific surface receptors (Gasser and Schifferli, 2004; Bianco et al., 2005), or by transfer of membrane receptors, as demonstrated for the chemokine receptor CCR5 (Mack et al., 2000) and the growth factor receptor EGFRvIII (Al-Nedawi et al., 2008). MV could function as messengers, being enriched in specific miRNA, mRNA, and proteins, to start an angiogenic program (Deregibus et al., 2007), to spread a danger signal through HMGB1 (Tucher et al., 2018) or to induce a developmental program (Ratajczak et al., 2006). In our experiments, we measured mRNA content of microglia released LPS/IFNγ-MV, but we cannot exclude additional transfer elements (such as membrane or signal proteins) from MV to glioma and TAM, responsible for the antitumor effects.

In addition to a modulation of TAM phenotype, we observed that LPS/IFNγ-MV affect glioma cell properties. We observed direct effects on glioma cell migration and invasion capability, and indirect effects on tumor cell viability, which requires the presence of parenchymal cells, likely microglia, as preferential target of microglia-derived EVs (Verderio et al., 2012). The effects of microglia-derived MV on GL261 cells could reflect the transfer of cell-donor information as reported for the first time for antigen-presenting cells that secrete exosomes able to stimulate T cell proliferation (Raposo et al., 1996) or, more recently in Alzheimer disease, for microglia released MV that exert toxicity on neurons (Joshi et al., 2014). In our experiments, EV-mediated reduction of tumor mass could be the cause of the EV-induced decreased neurotoxicity in the presence of glioma. However, we cannot exclude that EVs also directly target neurons, as shown by other authors (Antonucci et al., 2012; Chivet et al., 2014), but the lack of detectable PKH26-MV staining in extra tumoral regions of glioma bearing mice would suggest a direct or local paracrine effect. In addition, *in vitro* experiments demonstrated that microglia-derived MVs did not directly affect neuron viability.

The dimensional analyses of EVs extracted by primary mouse microglia and BV2 cell line reflect data reported on nervous system-released vesicles (Basso and Bonetto, 2016; Zappulli et al., 2016) and underline the similarity between primary microglia and cell lines, at least for EV dimension. Our TEM analysis shows that single particles have a spherical morphology; in addition to single EV, multiple aggregates are observed confirming size and shape already reported for EVs derived from microglial cells (Turola et al., 2012; Gouwens et al., 2018). In comparison with more physiological conditions (*in vivo* or *ex vivo*), cell lines and primary cultures of microglia display many differences in term of gene expression and response to stimuli (Horvath et al., 2008; Henn et al., 2009). However, there are evident advantages in using EVs derived from microglia cells lines, such as the possibility to obtain sufficient EVs for potential therapeutic application and the similar results obtained with MV from BV2 or from freshly isolated microglia encourage in this direction. However, a detailed analysis of the cargo differences in MV obtained from cell lines, cultured or freshly obtained microglial cells is lacking.

The choice to deliver MV *in situ* into the tumor region allowed us to use a relatively small amount of vesicles per infusion and to observe a direct effect on tumor mass. Different brain delivery of EVs, as into the cisterna magna (Casella et al., 2018), would give additional information on the effects of EVs in the contralateral hemisphere, for the multicellular targets of EVs. Additional delivery methods were tested for EVs, such as the intravenous (Alvarez-Erviti et al., 2011) and intra-nasal (Zhuang et al., 2011) approach. Intravenous administration permits indirect access of MV to the brain through the initial absorption in different cells (Laso-García et al., 2018). Intranasal delivery might reduce the final brain concentration due to lungs and gastrointestinal tract dilution. Nevertheless, both delivering routes would be easily reproduced in humans, while intratumor transfer would be only possible upon surgery procedures during glioma removal. For that reason, alternative EV injection routes, in glioma models, need further investigations. For brain tumor treatment, EVs have been tested to convey specific molecules such as small interference RNAs for TGF beta 1 and VEGF (Zhang et al., 2014; Yang et al., 2017), pro-apoptotic peptides (Ye et al., 2018), and chemotherapeutic drugs (Tang et al., 2012; Yang et al., 2015). EVs are preferred to artificial nanoparticles for their low toxicity (Lewinski et al., 2008; Shvedova et al., 2010).

To our knowledge, this is the first evidence that, in the context of a brain glioma, microglia-derived MVs can be used to transfer a cargo of molecular information that reach TAM restoring a neuroprotective phenotype, modulating their inflammatory state and re-establishing a homeostatic brain microenvironment.

## Author Contributions

AG, CS, and MC conceived the study and performed the experiments. GC provided technical support for primary mouse cultures and cannulae implantation. VN, AS, BR, and GR performed the DLS and LTS experiments, and analyzed the results. MR and GF performed the TEM experiments and analyzed the results. GRP and FG performed the NanoString experiments and analyzed the results. CL and MC conceived the study and wrote the manuscript. All authors reviewed and approved the final manuscript.

## Conflict of Interest Statement

The authors declare that the research was conducted in the absence of any commercial or financial relationships that could be construed as a potential conflict of interest.

## Footnotes

1 http://www.rstudio.com/

2 http://circe.med.uniroma1.it/fidea/
