# Supplementary material for: Microglia-Derived Microvesicles Affect Microglia Phenotype in Glioma
Source: Front Cell Neurosci. 2019 Feb 22;13:41. doi: 10.3389/fncel.2019.00041 (PMC6395438; doi:10.3389/fncel.2019.00041)
Supplement: Supplementary file 1 [file Table_1.pdf]

Supplementary Material:
"Microglia-derived microvesicles affect microglia phenotype in glioma"

1. Supplementary figures

Figure S1. (**A**) BV2 cells were untreated (C) or treated with LPS/IFNγ or IL 4 for 24 hours and NO released in cell medium was measured by Greiss reaction. Data are expressed as μM. N=3; **p<0,001 vs C. (**B**) BV2 cells were untreated (C) or treated with LPS/INFγ or IL4 for 24 h. Levels of Arg1 protein were assayed by Western blot. Data are the mean ± SEM expressed as arbitrary units, *p<0.003 *vs* C; N=3; actin was used as loading control. Representative blots are shown on the top; molecular weights are indicated on the right (KDa). (**C**) BV2 cells were untreated (C) or treated with LPS/IFNγ or IL 4 for 24 hours. Cell medium was collected to measure arginase activity. As a degree of arginase activity, the urea concentration was measured according to manufacturer kit instructions (MAK112, Sigma Aldrich). Data are expressed as mU/mL/μg proteins and are the mean ± SEM. N=3, *p = 0,002 vs C.

Figure S2. GL261 glioma cells were treated with Exo obtained from untreated microglia (C-MV), microglia treated with LPS/IFNγ (LPS/IFNγ-MV) and IL 4 (IL 4-MV) and a wound healing assay was performed. GL261 migration was measured 24 hour and 48 hour after treatment, data are expressed as mean percentage of wound healing area ± SE, N = 4, no statistical significance vs C-MV (One Way Analysis of Variance, Holm-Sidak method).

Figure S3. Tumor size in the brain of GL261-bearing mice treated with vehicle or MV obtained from BV2 cells untreated (C-MV), treated with LPS/IFNγ (LPS/IFNγ-MV) and IL 4 (IL 4-MV). Tumor size (in mm^3) was reported as mean ± SE, n=2 / experimental group.

Supplementary Methods.

Nitrite assay. NO production of BV2 cells untreated or treated with LPS/INFγ or IL4 was assessed by measuring nitrite accumulation in the culture medium by Griess Reagent Kit according to manufacturer instructions (Molecular Probes). The absorbance was measured at 570 nm in a spectrophotometric microplate reader (BioTek Instruments Inc, VT, USA).

Western blot analysis. BV2 cells were untreated or treated with LPS/IFNγ and IL 4 for 24 hours. Cells were lysed in RIPA buffer and protein amount was quantified by BCA assay (Pierce). Proteins were separated on 12% SDS-polyacrylamide gel electrophoresis and analyzed by Western immunoblot using Arg1 (sc-271430, Santa Cruz Biotechnology) 1:200 and Actin (Sigma Aldrich) 1:1000; HRP-tagged goat anti mouse and goat anti rabbit IgG were used as secondary antibodies (1:1000; Dako); the detection was performed through the chemiluminescence assay Immun-Star Western C Kit (Bio-Rad). Densitometric analysis was carried out with Quantity One software (Bio-Rad).

Arg1 activity. BV2 cells were untreated (C) or treated with LPS/IFNγ and IL 4 for 24 hours, cell were lysed in RIPA buffer and quantified by BCA assay (Pierce). Cell medium was collected to measure arginase activity. As a degree of the enzyme activity, the urea concentration was measured and the conversion into units of arginase activity was performed according to manufacturer kit instructions (MAK112, Sigma Aldrich). One unit (U) of arginase activity is defined as the amount of enzyme that catalyzes the formation of 1 μmol urea/min.
