# Supplementary figures and images for: Microglia-Derived Microvesicles Affect Microglia Phenotype in Glioma
Source: Front Cell Neurosci. 2019 Feb 22;13:41. doi: 10.3389/fncel.2019.00041 (PMC6395438; doi:10.3389/fncel.2019.00041)

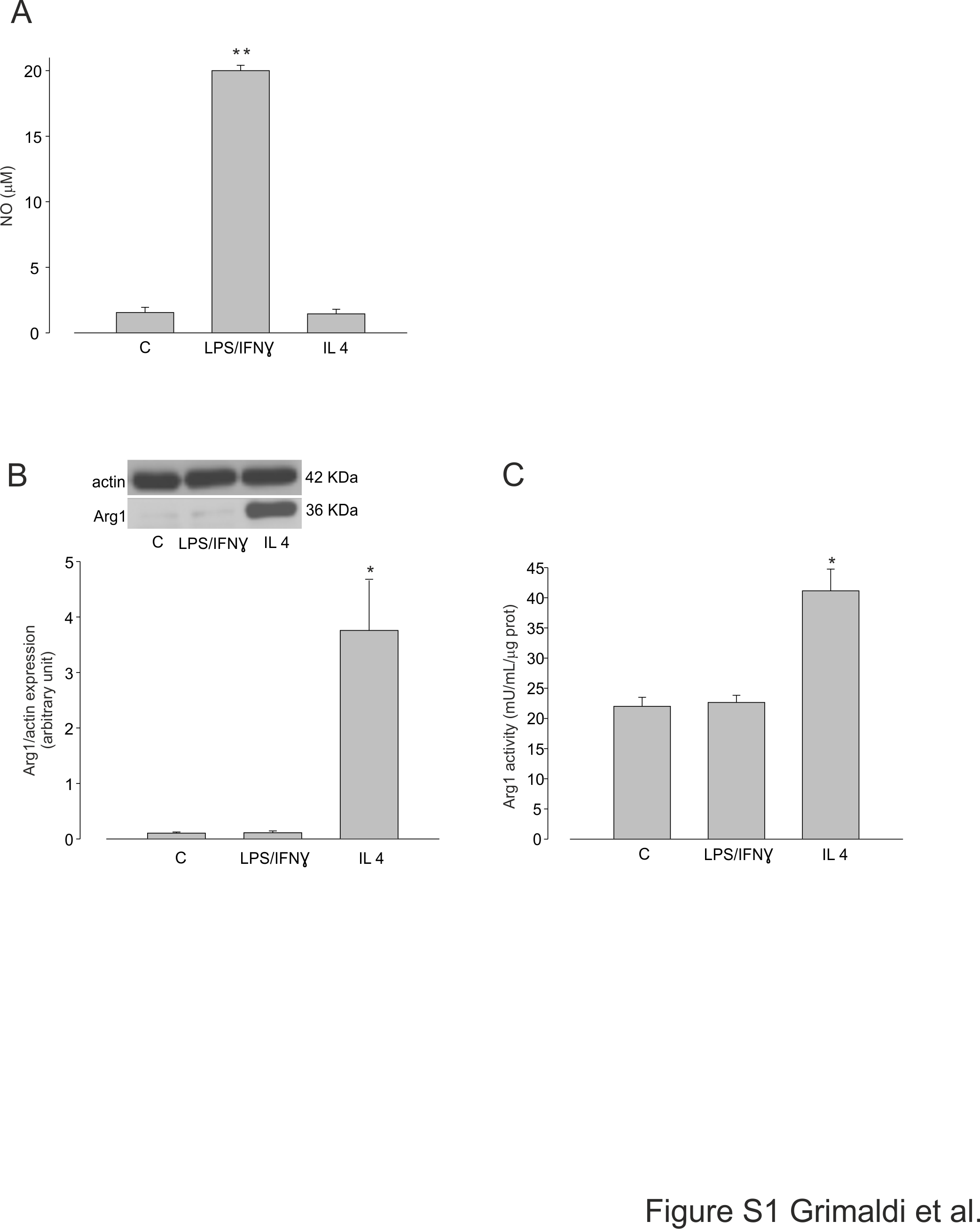

Supplement: Supplementary file 2 [file Image_1.tif]

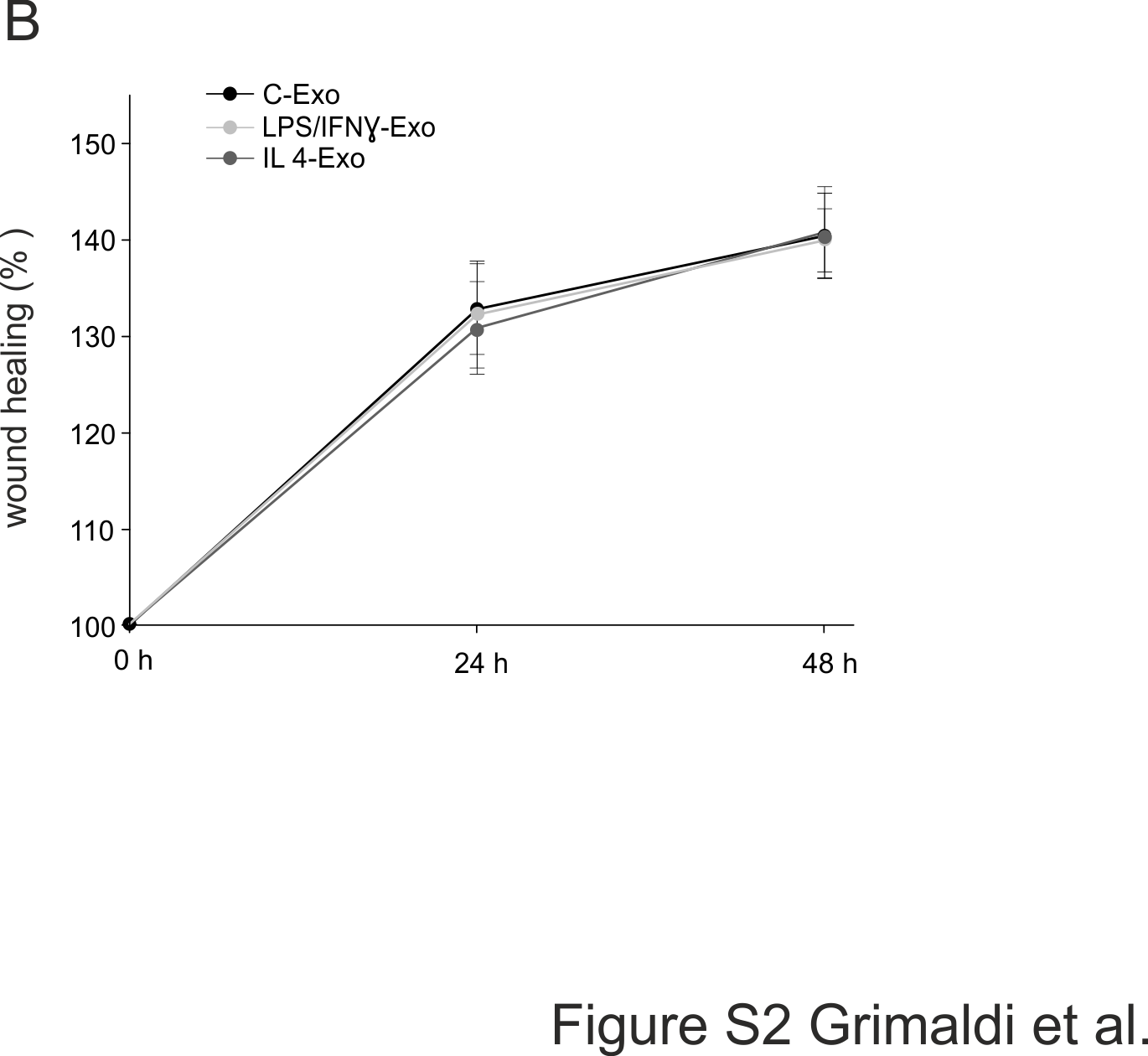

Supplement: Supplementary file 3 [file Image_2.tif]

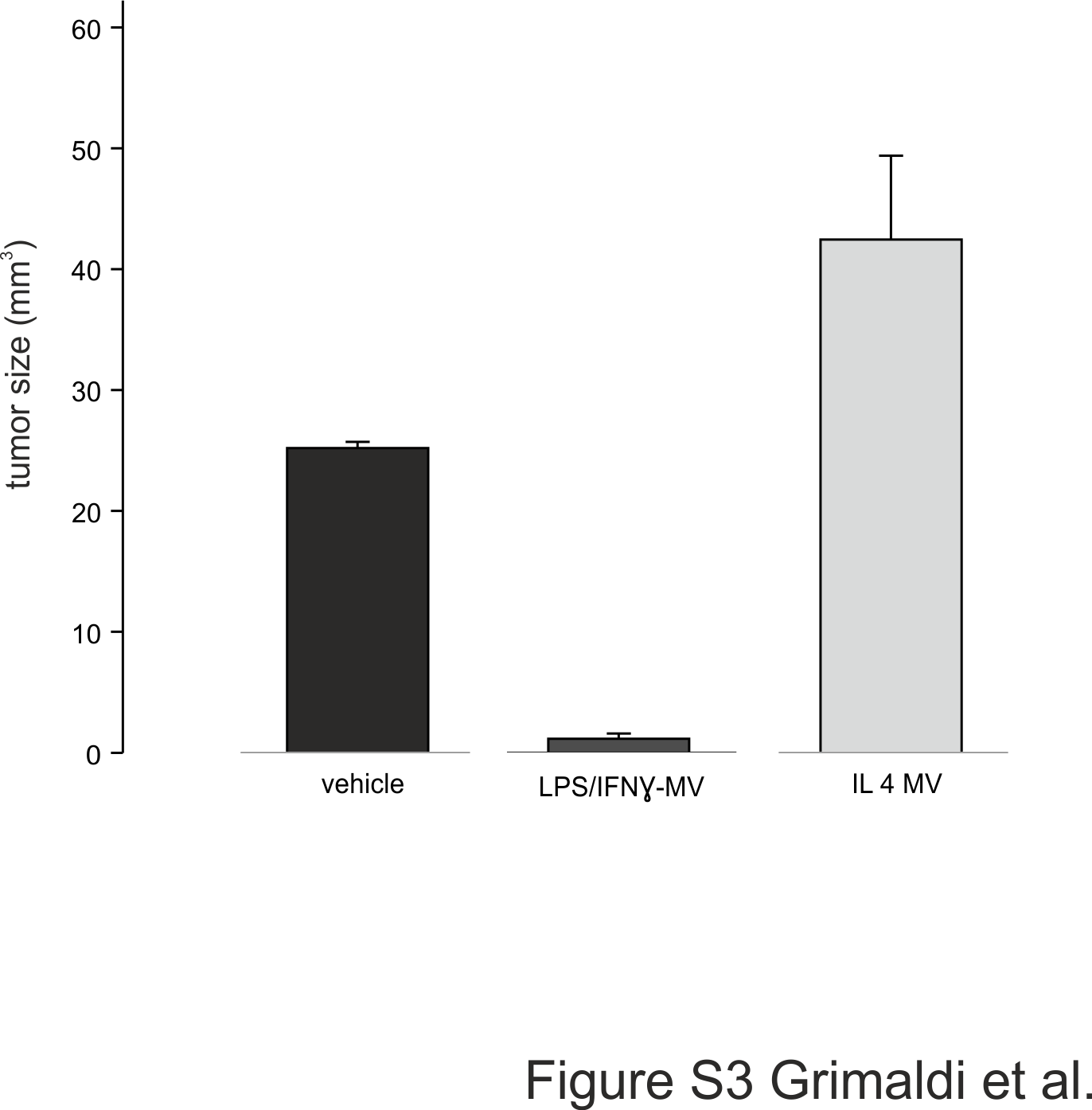

Supplement: Supplementary file 4 [file Image_3.tif]
